# Electron spin relaxations of phosphorus donors in bulk silicon under large electric field

**DOI:** 10.1038/s41598-019-39613-4

**Published:** 2019-02-27

**Authors:** Daniel K. Park, Sejun Park, Hyejung Jee, Soonchil Lee

**Affiliations:** 1 Department of Physics, Korea Advanced Institute of Science and Technology, Daejeon, 34141 Korea; 2 Present Address: School of Electrical Engineering, Korea Advanced Institute of Science and Technology, Daejeon, 34141 Korea; 3 Present Address: Department of Physics, Imperial College London, London, SW7 2BW United Kingdom

## Abstract

Modulation of donor electron wavefunction via electric fields is vital to quantum computing architectures based on donor spins in silicon. For practical and scalable applications, the donor-based qubits must retain sufficiently long coherence times in any realistic experimental conditions. Here, we present pulsed electron spin resonance studies on the longitudinal (*T*_1_) and transverse (*T*_2_) relaxation times of phosphorus donors in bulk silicon with various electric field strengths up to near avalanche breakdown in high magnetic fields of about 1.2 T and low temperatures of about 8 K. We find that the *T*_1_ relaxation time is significantly reduced under large electric fields due to electric current, and *T*_2_ is affected as the *T*_1_ process can dominate decoherence. Furthermore, we show that the magnetoresistance effect in silicon can be exploited as a means to combat the reduction in the coherence times. While qubit coherence times must be much longer than quantum gate times, electrically accelerated *T*_1_ can be found useful when qubit state initialization relies on thermal equilibration.

## Introduction

Phosphorus donor spins in silicon (Si:P) are promising candidates for encoding quantum information due to outstanding coherence times and the availability of the mature semiconductor industry. Since the quantum computing architecture based on donor spins in silicon was proposed by Kane^1^, many significant milestones, such as extending qubit coherence times via silicon-28 isotope enrichment^2–6^, high-fidelity control and readout of single donor spins^7–10^, and resonance frequency tuning using electric fields induced Stark shift for the qubit-selective control^11,12^, have been achieved. Also, several device designs within the donor-based framework have been proposed^13–15^ to enable topological quantum error correction. However, realizing two-qubit quantum operations using the exchange interactions as originally proposed by Kane in a scalable manner remains challenging due to the extreme sensitivity of the interaction strength to the donor displacement. To relax the requirement on the spatial precision, an architecture that exploits electric dipole interactions was recently proposed^16^. In this approach, the qubit is defined using the flip-flop energy splitting of the nuclear and electron spin states, and is called the flip-flop qubit. One and two qubit gate implementations require shifting the donor electron wavefunction to the ionization point, where the electron is shared halfway between donor and Si/SiO_2_ interface, via electric fields. This scheme allows for the larger inter-qubit spacing than the exchange-based method, which yields sufficient room to place classical control and readout components^16^. After all, the application of electric fields is ubiquitous in various proposals for donor-based quantum information processing. On the other hand, theoretical analyses of the single-donor flip-flop qubit with reasonable experimental parameters predicted considerable decrease of the qubit *T*_1_ relaxation time due to strong interaction with phonon-induced deformation potentials and the nontrivial valley-related characteristics of the electron-phonon interaction and the involved electronic states^17^. Yet, the effect of electric fields near ionization on the *T*_1_ and *T*_2_ times of donor spins in silicon with higher qubit densities is not clear-cut.

This work reports a detailed experimental study on the spin relaxation times of phosphorus (^31^P) donor electrons in bulk silicon under electric fields (*E*_0_) ranging up to near avalanche breakdown triggered by impact ionization using pulsed electron spin resonance (ESR). Two Si:P wafers with donor concentrations of about 10^14^–10^15^ P/cm^3^ are tested in the external static magnetic field (*B*_0_) strength of about 1.2 T and at temperatures near 8 K for good compromise between the detection sensitivity and the *T*_1_ relaxation time in the absence of electric field. The key findings are summarized as follows. The *T*_1_ relaxation time changes dramatically under strong electric field near (but before) the avalanche breakdown, and the coherence time *T*_2_ is limited by *T*_1_. This is attributed to the electric current (I) in the bulk silicon. However, due to magnetoresistance in silicon^18,19^, the amount of electric current varies with the orientation of the electric field with respect to the magnetic field. Therefore, the reduction in the relaxation times can be minimized by carefully determining the orientation.

## Results

**Figure 1:**
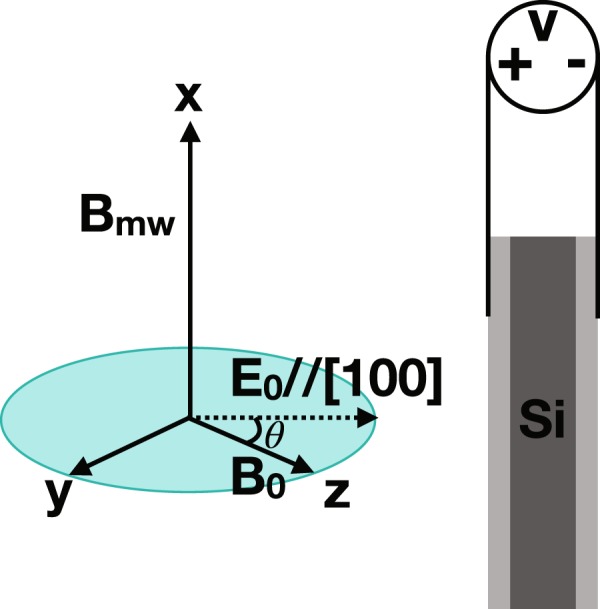
Schematic of the Si:P wafer (dark gray) with aluminum (light gray) sputtered on two faces of the wafer (not in scale) and its orientation with respect to external fields. The electric field, *E*_0_, is formed between the metal plates by applying DC voltage. The sample is inserted in the static magnetic field, *B*_0_, in such a position that the direction of the electric field is coplanar to the magnetic field. The microwave field, *B*_mw_, for controlling electron spins points perpendicular to both *E*_0_ and *B*_0_. The coordinate frame on the left is arbitrarily defined for convenience.

The spin relaxation times of the donor-bound electrons are measured from two Si:P wafers, A and B, with phosphorus concentrations of 2.2 × 10^14^−4.9 × 10^15^ P/cm^3^ and 3.5 × 10^14^−6.5 × 10^14^ P/cm^3^, respectively, using a Q-band ESR spectrometer (see Methods for details). These concentrations correspond to about 59 to 166 nm inter-donor distance assuming uniform distribution. The electric field is formed along the [100] crystal orientation by applying voltage between two 50-nm-thick aluminum plates sputtered on each face of the wafer. The direction of *E*_0_ is coplanar to the external static magnetic field, and perpendicular to the oscillating microwave field (*B*_*mw*_) as shown in Fig. 1.

**Figure 2:**
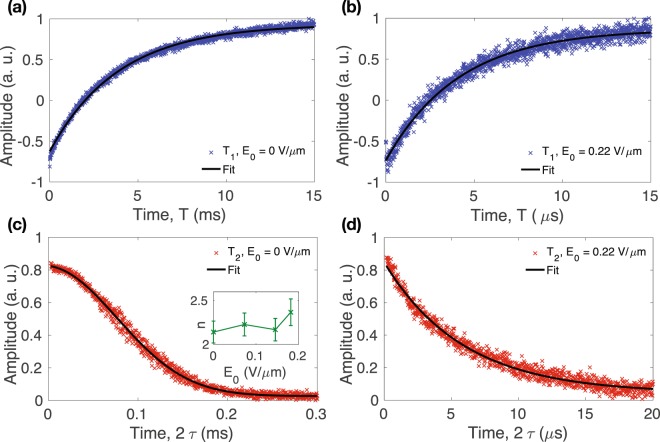
Examples of the electron spin echo decay to measure *T*_1_ (**a**) without electric field, and (**b**) with *E*_0_ = 0.22 V/*μ*m, and to measure *T*_2_ (**c**) without electric field, and (**d**) with *E*_0_ = 0.22 V/*μ*m for sample A at around 1.2 T and 8 K. The solid line in (**c**) is the fitting to *s*(2*τ*) = exp[−(2*τ*/*T*_2*a*_)^*n*^ − 2*τ*/*T*_2*b*_], and the inset shows the fitted values of *n* with the error bars representing the fitting error. The data in (**a**,**b** and **d**) are fitted using a single exponential decay curve.

For both samples, the electron spin echo signal decays in the *T*_1_ measurement experiments are single exponential regardless of the magnitude of the electric field. On the other hand, when *E*_0_ = 0, the *T*_2_ decay curve is better described by *s*(2*τ*) = exp[−(2*τ*/*T*_2*a*_)^*n*^ − 2*τ*/*T*_2*b*_], where *s*(2*τ*) is the normalized electron spin echo signal with the interpulse delay *τ*^20^. However, we found that as the *E*_0_ value reaches certain regime, the *T*_2_ decay becomes a single exponential. In our measurements, the *E*_0_ values from which the coherence decay curves are single exponential are 0.22 V/*μ*m and 0.13 V/*μ*m for sample A and B, respectively. Moreover, at these points, the *T*_1_ relaxation times are significantly reduced. Figure 2 shows examples of electron spin signal decay from the *T*_1_ [(a) and (b)] and *T*_2_ [(c) and (d)] measurements for sample A with *E*_0_ = 0 and 0.22 V/*μ*m at *B*_0_ = 1.2 T and 8 K. The inset in Fig. 2(c) shows that *n* estimated from fitting for several values of *E*_0_ in the non-single exponential regime ranges between two to three, agreeing with previously reported values attributed to nuclear-induced spectral diffusion^3,20^. The large difference between the time scale (horizontal axis) of the figures on the left [(a) and (c)] and on the right [(b) and (d)] demonstrates the substantial increase of the relaxation rates when the electric field is turned on.

**Figure 3:**
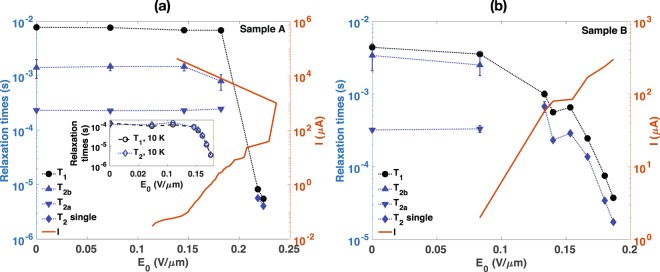
The electric field dependence of electron spin relaxation rates (left y-axis) for the Si:P sample (**a**) A and (**b**) B at near 1.2 T and 8 K. The inset in (**a**) shows the relaxation rates as a function of *E*_0_ at 10 K. The circles represent the *T*_1_ time, two triangle symbols are obtained from the non-exponential signal decay in the *T*_2_ measurement, and the diamonds represent the *T*_2_ time in the large electric field regime where the signal decay becomes single exponential. The solid lines (right y-axis) represent the electric current between two aluminum plates on the faces of the silicon wafer as a function of *E*_0_. In (**a**), the avalanche breakdown occurs around 0.24 V/*μ*m as indicated by the discontinuity in the current-electric field curve. The error bars represent fitting errors, and are smaller than the data symbol when not visible.

The relationship between *E*_0_ and the relaxation times is plotted in Fig. 3. For A, *T*_1_ is reduced by about three orders of magnitude at *E*_0_ ≥ 0.22 V/*μm*. The coherence decays fit well to single exponential in this regime, and *T*_2_ appears to be limited by *T*_1_. The solid line represents the magnitude of electric current between the metal plates, and shows the avalanche breakdown, a sudden transition to a low-resistance state, occurs at about 0.24 V/*μ*m for this sample. Thus, *E*_0_ could not be increased beyond this point. Interestingly, the relaxation times change dramatically even though the current is about two orders of magnitude smaller than that at the breakdown. The inset in Fig. 3(a) shows the relaxation times in A as a function of *E*_0_ at 10 K to demonstrate that the effect persists in a different temperature. In this temperature and donor concentration, *T*_2_ is limited by *T*_1_ as the *T*_1_ process dominates decoherence^6^. The relaxation times decrease rapidly as *E*_0_ is increased beyond 0.15 V/*μ*m, similar to the behaviour observed at 8 K. The electric field dependence of the relaxation times is qualitatively confirmed with the sample B as shown in Fig. 3(b). Both *T*_1_ and *T*_2_ do not exhibit noticeable changes until *E*_0_ is increased up to about 0.08 V/*μ*m. But from beyond this point, *T*_1_ undergoes about two orders of magnitude reduction as *E*_0_ is increased up to about 0.19 V/*μm*. Meanwhile, the *T*_2_ decay converges to single exponential, and the spin coherence time appears to be limited by the *T*_1_ process in this regime. The experimental data provide clear evidence that the longitudinal relaxation time of electron spins in Si:P is reduced substantially even when the applied electric field is smaller than the breakdown field. On the other hand, the impact of the electric field on *T*_2_ independent from the *T*_1_ process is uncertain. The electric field effects on the spin relaxation times of the donor-bound electron in bulk silicon implies that the operating conditions of the Si:P quantum devices with higher donor densities can be limited due to accelerated decoherence. Hereinafter, we focus on the effect of the electric field on *T*_1_.

**Figure 4:**
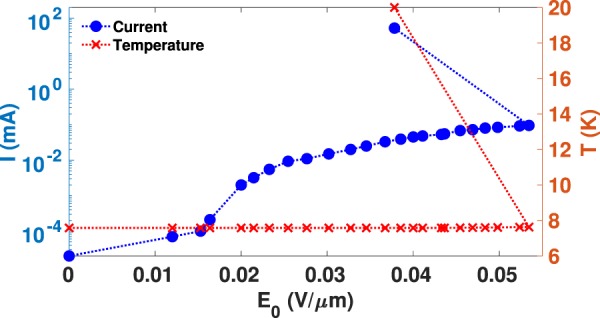
The electric current between two aluminum plates on the faces of the silicon sample A (left y-axis) and the sample temperature (right y-axis) as a function of the electric field strength, *E*_0_. The avalanche breakdown is observed by the discontinuity in the current-electric field curve.

In the temperature range in which the measurements are conducted, the strong temperature dependence of *T*_1_ is known to exist due to spin-phonon relaxation process^21,22^. As a first step towards understanding the source of the electric field dependence of the relaxation times, we measured the sample temperature and the electric current in a Si:P wafer between two metal plates with respect to *E*_0_ (see Methods). The measurement results depicted in Fig. 4 show that the sample temperature remains nearly constant until the breakdown field, at which the resistivity of the sample abruptly drops, is reached. Thus, the dramatic *T*_1_ reduction in our measurements cannot be explained by the change in the sample temperature.

**Figure 5:**
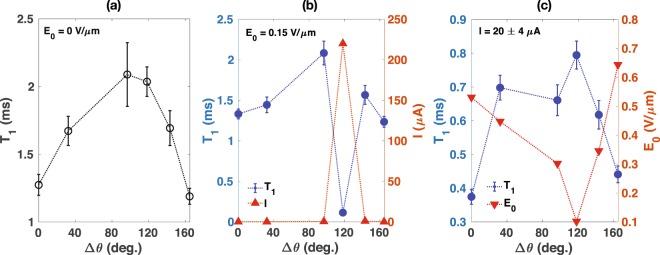
The *T*_1_ relaxation times for the sample B (left y-axis) as a function of an angle between *E*_0_ and *B*_0_ when (**a**) *E*_0_ = 0, (**b**) *E*_0_ = 0.15 V/*μ*m, and (**c**) *I* = 20 ± 4 *μ*A. Δ*θ* corresponds to the change of the angle between *E*_0_ and *B*_0_. For a fixed value of *E*_0_, the electric current varies with the orientation, and vice versa. The anisotropy of the current and *E*_0_ are presented in (**b**) and (**c**), respectively (right y-axis). The vertical error bars represent fitting errors. The uncertainty in Δ*θ* is smaller than the width of the symbols.

Next, we experimentally investigated the anisotropy of *T*_1_ in the crystal orientation with respect to *B*_0_ using B, the sample with more uniform donor distribution. In this study, the silicon crystal, and hence the direction of *E*_0_, were rotated around the x-axis defined in Fig. 1 while the direction of *B*_0_ was fixed. For each orientation, we conducted *T*_1_ measurements with *E*_0_ = 0, 0.15 V/*μ*m, and at constant electric current of 20 ± 4 *μA* measured in the same direction as *E*_0_. To maintain the constant electric current at each orientation, the strength of *E*_0_ is adjusted by controlling the DC voltage. The results are shown in Fig. 5. Note that the independent variable in the figure (Δ*θ*) corresponds to the change of the angle between *E*_0_ and *B*_0_ from the reference angle determined by the initial sample placement in the magnetic field. The *T*_1_ relaxation time is observed to be anisotropic in the crystal orientation, and hence the direction of *E*_0_, with respect to *B*_0_ in all experiments. For a fixed amount of *E*_0_, we observed that the electric current in [100] direction also varied with respect to the orientation between *E*_0_ and *B*_0_. As shown in Fig. 5(b), when *E*_0_ = 0.15 V/*μ*m, the current is maximum near Δ*θ* = 120°. Interestingly, *T*_1_ at this orientation is about an order of magnitudes smaller than that at other orientations. The anisotropic *T*_1_ is also observed when the current is fixed at about 20 *μ*A as illustrated in Fig. 5(c). In this case, however, the qualitative feature of the orientation dependence of *T*_1_ resembles that of the orientation dependence observed when the electric field is turned off (Fig. 5(a)), except the relaxation times at each point are reduced by approximately a factor of three.

## Discussion

The spin relaxation anisotropy without the electric field displayed in Fig. 5(a) is consistent with the previous results attributed to a modulation of the electronic *g* factor by acoustic phonons^23–25^. The previous studies show that the *T*_1_ relaxation time is the longest when *B*_0_ is aligned with [100] axis of the silicon crystal^25^. Thus, in our experimental data, the [100] axis lies within the range of Δ*θ* = 96° to 118°.

Figure 5(b) shows that the electric current in the wafer between two metal plates is the largest when *B*_0_ is aligned with [100] axis, along which *E*_0_ is applied. The variation of the electric current with respect to the angle between *E*_0_ and *B*_0_ for a fixed electric field strength is consistent with the effect of magnetoresistance (MR), defined as [*R*(*B*) − *R*(0)]/*R*(0), where *R*(*B*) is the resistance in magnetic field *B*. The phosphorus density of sample B is just within the low doping regime where the MR for the perpendicular orientation (*B*_0_ ⊥ *E*_0_) is large^26^. For Si:P, the MR effect persists for the longitudinal orientation, i.e., ${B}_{0}\parallel {E}_{0}$, since the Lorentz force can still deflect the carriers as the trajectories are distorted due to the random distribution of donors. Nevertheless, the longitudinal MR is known to be much smaller than the perpendicular MR^26,27^. Therefore, the large electric current at a specific orientation (Δ*θ* ≈ 118°) can be interpreted as a consequence of the minimum MR since *E*_0_ is parallel to *B*_0_.

When *E*_0_ = 0.15 V/*μ*m, although the electric field is uniformly raised for all orientations, the *T*_1_ time is significantly reduced only when the electric current is high. On the other hand, Fig. 5(c) shows that despite the large variation of *E*_0_ with respect to the orientation, all relaxation times are about a factor of three smaller than the relaxation times at the same orientation without the electric field. These experimental results suggest that the acceleration of the longitudinal relaxation rate at strong electric field is related to the rise of the electric current in the Si:P crystal rather than the strength of the electric field.

The quality of the electron spin qubits in Si:P can be degraded considerably when large electric field is needed, due to the increased decoherence rate. In order to enhance the utility and the flexibility of the donor-based spin qubits, it is desirable to extend the high-fidelity qubit operating range to strong electric fields. Moreover, there exists an architectural proposal that demands the application of strong electric fields so that the electron spins are manipulated near the ionization point^16^. Therefore, finding strategies to circumvent the escalation of the spin relaxation rates is critical. From above experimental studies, we found that the electric current, rather than the electric field, is responsible for the rapid change in *T*_1_. Then we experimentally verified that MR yields the electric current anisotropy in the orientation of *E*_0_ with respect to the external magnetic field. Therefore, if the angle between *E*_0_ and *B*_0_ is chosen properly, the electric current for a given *E*_0_ strength, and hence the reduction in the *T*_1_ time can be minimized. In particular, the electric field should not be aligned with the magnetic field. For instance, Fig. 5(b,c) illustrate that when *E*_0_ is nearly parallel to *B*_0_ (Δ*θ* ≈ 120°), *T*_1_ is about 0.1 ms for *E*_0_ = 0.15 V/*μ*m, while when *E*_0_ is nearly perpendicular to *B*_0_ (Δ*θ* ≈ 40°), *T*_1_ is about 0.7 ms for *E*_0_ = 0.45 V/*μ*m. Thus, in this example data, the *T*_1_ time at one orientation can be about seven times longer than that at another orientation, although about three times larger *E*_0_ is used. Recall that in the absence of *E*_0_, the *T*_1_ time is the longest when *B*_0_ is applied along the [100] direction. Thus, we speculate that further *T*_1_ optimization is possible by using a silicon wafer grown in a direction such that the direction of *B*_0_ can be parallel to [100], but perpendicular to *E*_0_.

In summary, the *T*_1_ relaxation rate of the phosphorus donor electron spins in bulk silicon with low dopant concentration can be increased significantly under large electric fields due to electric current in the sample. The coherence time is also shortened as it is upper-bounded by *T*_1_. On the other hand, the amount of electric current is anisotropic in the *E*_0_ orientation with respect to the external magnetic field due to the MR effect. Thus, the reduction in the relaxation rates for a fixed electric field strength can be minimized by choosing the appropriate orientation. Although the decoherence rate must be minimized during quantum gate operations, the fast *T*_1_ relaxation can be exploited in a special instance. Namely, when the qubit initialization method relies on thermal equilibration, the fast relaxation rate is favoured for resetting the qubits. Furthermore, the ability to engineer the *T*_1_ relaxation rate can be useful for dynamic nuclear polarization since the increased *T*_1_ rate allows for the faster polarization of nuclear spins at the cost of the higher microwave pulse power for saturating the electron spin transitions^28^. Future work could extend the range of the experimental conditions, such as the magnetic field strength, the temperature, and the donor density. Our results also motivate further studies on the nuclear spin relaxation rates, as well as the case of the single donor spins in large electric fields.

## Methods

### Sample preparation

Two commercially purchased phosphorus doped (100)-silicon wafers with natural abundance (4.7% of ^29^Si) are used throughout the experiments. The first wafer (A) is quoted with the room temperature resistivity of 1–20 Ω⋅cm (about 2.2 × 10^14^−4.9 × 10^15^ P/cm^3^), and the thickness of 275 *μ*m. The second wafer (B) has the room temperature resistivity of 7–13 Ω⋅cm (about 3.5 × 10^14^−6.5 × 10^14^ P/cm^3^), and is 300 *μ*m thick. The wafers are cut to a size of approximately 1 × 15 mm to fit in a standard Q-band ESR tube.

### Electron spin resonance measurements

All ESR experiments were carried out at Korea Basic Science Institute (KBSI) in Seoul, Korea. 34 GHz Q-band pulsed ESR data were obtained on a Bruker Elexsys E580 spectrometer using an EN5107D2 resonator. Cryogenic temperatures were achieved with an Oxford CF-935 cryostat and an Oxford ITC temperature controller.

### Relaxation times measurements

*T*_1_ is measured via inversion recovery experiment, and *T*_2_ is measured via Hahn echo decay experiment. The pulse sequence for the inversion recovery experiment can be expressed as *π*−*T*−*π*/2−*τ*−*π*−*τ*−echo detection. The delay, *T*, after the first *π* pulse was varied while *τ* was fixed, and the amplitude of the primary echo signal formed by the second and third pulses was measured. The pulse sequence for the Hahn echo decay experiment is *π*/2−*τ*−*π*−*τ*−echo detection, and the amplitude of the echo signal was measured as a function of the delay, *τ*. The *π*/2 and *π* pulse lengths were 16 and 32 ns, respectively, in both experiments.

### Current-voltage measurements

The current-voltage relations are measured using two multimeters (Fluke 287 True-RMS), each connected in parallel and in series with the silicon wafer for measuring voltage and current, respectively.

### Sample temperature measurements

The sample temperature dependence on the electric field strength was measured with an Si:P piece cut from A to an approximate size of 1 cm^2^. A calibrated temperature sensor (Lakeshore DT-470-CU-13) was attached on top of the aluminum on one face of the wafer, and the other face of the wafer was in contact with the copper heat-sink of a low temperature probe. Then the probe was cooled using a helium-flow cryostat. The current was measured using a multimeter while external DC voltage was applied.

## Data Availability

The datasets generated during and/or analysed during the current study are available from the corresponding author on reasonable request.
